# New York State Lunatic Asylum

**Published:** 1890-02

**Authors:** 


THE PROPOSITION comes from the managers of the New York State Lunatic Asylum, at Utica, to change the name of that Institution to the Utica State Hospital. We hope the Legislature will act promptly and favorably on the measure if it should crystallize into the form of a bill. There is apparently no good reason why the hospitals where diseases of the brain and nervous system are treated should be called asylums; certainly it would be as pertinent to call any and all hospitals asylums as these.

The humane stand taken by the Superintendent of the Utica Asylum in this and other matters relating to the management of his institution, are to be commended, and we trust he will receive the cordial support of the profession in carrying forward all measures of reform regarding the care and treatment of the insane. The Utica *Daily Press*, in its issue of January 16, 1890, speaks editorially upon this subject in a most liberal and proper spirit, and its wise words deserve to be well pondered by all interested in the considerate betterment of the environment of the class of invalids in question.

